# The transmembrane domains of the type III secretion system effector Tir are involved in its secretion and cellular activities

**DOI:** 10.3389/fcimb.2023.1103552

**Published:** 2023-02-14

**Authors:** Dor Braverman, Jenia Gershberg, Neta Sal-Man

**Affiliations:** The Shraga Segal Department of Microbiology, Immunology, and Genetics, Faculty of Health Sciences, Ben-Gurion University of the Negev, Beer-Sheva, Israel

**Keywords:** bacterial virulence, EPEC, transmembrane domains, type III secretion system, Tir

## Abstract

**Introduction:**

Enteropathogenic *Escherichia coli* (EPEC) is a diarrheagenic pathogen and one of the major causes of gastrointestinal illness in developing countries. EPEC, similar to many other Gram-negative bacterial pathogens, possesses essential virulence machinery called the type III secretion system (T3SS) that enables the injection of effector proteins from the bacteria into the host cytoplasm. Of these, the translocated intimin receptor (Tir) is the first effector to be injected, and its activity is essential for the formation of attaching and effacing lesions, the hallmark of EPEC colonization. Tir belongs to a unique group of transmembrane domain (TMD)-containing secreted proteins, which have two conflicting destination indications, one for bacterial membrane integration and another for protein secretion. In this study, we examined whether TMDs participate in the secretion, translocation, and function of Tir in host cells.

**Methods:**

We created Tir TMD variants with the original or alternative TMD sequence.

**Results:**

We found that the C-terminal TMD of Tir (TMD2) is critical for the ability of Tir to escape integration into the bacterial membrane. However, the TMD sequence was not by itself sufficient and its effect was context-dependent. Moreover, the N-terminal TMD of Tir (TMD1) was important for the postsecretion function of Tir at the host cell.

**Discussion:**

Taken together, our study further supports the hypothesis that the TMD sequences of translocated proteins encode information crucial for protein secretion and their postsecretion function.

## Introduction

Enteropathogenic *Escherichia coli* (EPEC) is a Gram-negative, facultative anaerobic, rod-shaped bacterium that infects epithelial cells in the gastrointestinal tract. EPEC is a major cause of infantile diarrhea in developing countries that has recently re-emerged and been reported in various outbreaks in Oceania, East Asia, and Northern Europe (Kaper et al., 2004; Canizalez-Roman et al., 2016; Lee et al., 2022). The hallmark of EPEC colonization is the formation of distinct histopathological lesions termed “attaching and effacing” (A/E) lesions. Other members of the A/E family include the human pathogen enterohemorrhagic *E. coli* (EHEC), which causes hemorrhagic colitis and pediatric kidney failure, and the mouse pathogen *Citrobacter rodentium*, which is used in animal models (Gaytan et al., 2016). A/E lesions are characterized by destruction of the intestinal microvilli, intimate bacterial attachment to the plasma membrane of enterocytes, and a cytoskeletal rearrangement that results in the formation of dense actin filaments beneath adherent bacteria, termed pedestals (Moon et al., 1983; Wong et al., 2011). The development of A/E lesions involves three sequential stages: (i) initial bacterial adherence, (ii) cellular signal transduction, and (iii) intimate attachment (Donnenberg and Kaper, 1992).

To allow bacterial adherence and interference with cellular signal transduction, EPEC uses a specialized protein secretion complex called the type III secretion system (T3SS). The T3SS is a syringe-shaped nanomachine that spans the bacterial membranes and extends to the host cell membrane, where it forms a pore that allows the translocation of various effector proteins across the plasma membrane and into the host cell cytoplasm. These effectors subvert key cellular processes (such as immune response, signal transduction, vesicle transport, and cytoskeletal dynamics) to promote bacterial replication, survival, and transmission. In A/E pathogens, the T3SS is encoded on a 35-kbp chromosomal pathogenicity island called the locus of enterocyte effacement (LEE). Type III secretion (T3S) activity is a tightly regulated process that is divided into the secretion of three substrate groups to ensure proper assembly and timely secretion. These groups comprise early (needle and inner rod proteins), intermediate (translocators), and late (effectors) substrates (Gaytan et al., 2016; Deng et al., 2017).

**Figure 1:**
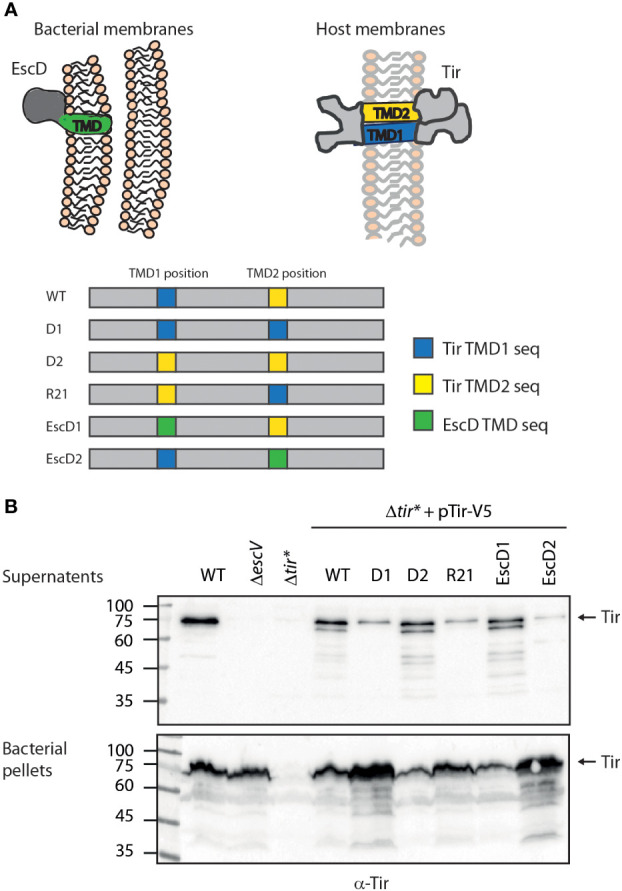
Tir TMD2 is critical for protein secretion. **(A)** Schematic illustration of Tir folding across the host membrane and the organization of TMDs in the WT and TMD-exchanged Tir variants. **(B)** Protein secretion profiles of EPEC strains grown under T3SS-inducing conditions: WT, Δ*escV*, Δtir*, and Δtir* carrying the pTir_wt_-V5 and Tir TMD-exchanged variants. The secreted fractions were normalized, concentrated from the supernatants of bacterial cultures, and analyzed by western blotting with an anti-Tir antibody. Bacterial pellets were analyzed to verify Tir expression. An arrow at the right of the gels indicates full-length Tir.

The first effector to be translocated into the host cell is the translocated intimin receptor (Tir). The delivery of a self-receptor to the host cell by the bacterium is considered a novel infection strategy that has only been reported in A/E pathogens. Tir adopts a hairpin loop topology on the plasma membrane, with cytosolic N and C termini and two helical transmembrane domains (TMDs) traversing the host membrane (**Figure 1A**). The central loop is exposed to the extracellular environment and interacts with the C terminus of intimin, a bacterial outer membrane adhesin presented on the EPEC membrane, with high affinity (Kd ~10 nM for EPEC). This interaction facilitates the tight attachment between the bacteria and the host (Lai et al., 2013). Upon Tir–intimin interaction, intimin dimerizes and leads to Tir clustering. This stimulates phosphorylation of Tir at various residues in its C terminus domain (Touze et al., 2004). While the role of serine/threonine phosphorylation in Tir is not fully understood, it is well established that tyrosine phosphorylation of Tir is required for the rearrangement of filamentous actin into pedestal structures, leading to the formation of A/E lesions (Lai et al., 2013; Gaytan et al., 2016).

Tir belongs to a unique group of secreted proteins that contain at least one TMD. Specifically, in EPEC, two translocator proteins, EspB and EspD, and two effectors, Tir and EpsZ, are TMD-containing secreted proteins. These proteins have, by definition, a targeting conflict because their sequence contains signals for two incompatible pathways. On the one hand, they contain an N-terminal T3SS signal that guides them for export through the T3SS and, on the other, they have a TMD signal that guides them to the bacterial membrane (Wagner et al., 2018). This conflict requires targeting discrimination, which ensures that, post-translation, the TMD-containing secreted proteins can escape the bacterial membrane integration mechanism and remain soluble at the cytoplasm until their timely secretion. While the mechanism underlying this targeting discrimination remains largely unknown, recent studies have suggested that a major factor is the moderate hydrophobicity of the TMDs of these secreted proteins, which is sufficient for integration into eukaryotic cell membranes but is not recognized by the bacterial signal recognition particle (Krampen et al., 2018). This was recently demonstrated by our group, where replacement of the TMDs of EPEC translocators (EspB and EspD) with a more hydrophobic sequence resulted in their mislocalization into the bacterial membrane and abolished their secretion (Gershberg et al., 2021). In addition, the ability of Tir to escape the co-translational pathway and membrane integration mechanism was previously suggested to be mediated through interaction with its chaperone, CesT (Elliott et al., 1999). Although the minimal chaperone binding domain (CBD) of Tir was found to be located at residues 35–77, together with an additional binding region at the C terminus of Tir (Little and Coombes, 2018), it was reported that T3SS chaperones might also interact with translocator and effector TMDs to mask them from the signal recognition particle and prevent erroneous targeting to the bacterial membrane (Krampen et al., 2018).

In the present study, we expanded our research to Tir, as an additional TMD-containing secreted protein, and examined the role of its TMDs in its secretion, translocation, and post-translocation functions. Moreover, we studied whether the context of the specific TMDs affects these properties. Our results support the previous observation that the TMD sequences of TMD-containing secreted proteins are crucial for protein secretion, as well as the importance of their context within the protein sequence. Moreover, our data suggested that, post-secretion, the TMDs affect Tir function at the host-cell membrane. Specifically, it appears that the C-terminal TMD of Tir (TMD2) is important for proper secretion while the N-terminal TMD (TMD1) is required for the post secretion function of Tir.

## Materials and methods

### Bacterial strains

All strains and plasmids used in this study are listed in **Table S1**. Wild-type EPEC O127:H6 strain E2348/69 [streptomycin-resistant] and EPEC null mutants (Δ*escN*, Δ*escV*, and Δtir*) were used to assess the T3SS and translocation activities. The Δtir* strain was a generous gift from Prof. Ilan Rosenshine (Hebrew University of Jerusalem, Israel) and contains a deletion of the 79–873-bp region of Tir. This modified strain has nonfunctional Tir but expresses normal levels of CesT, which is critical for the expression of virulence genes *via* inhibition of CsrA (Elbaz et al., 2019). *E. coli* DH10B was used for plasmid handling and *E. coli* strain BL21 (λDE3) was used for protein expression. The *E. coli* strains were grown at 37°C in Luria-Bertani (LB) broth (Sigma) supplemented with the appropriate antibiotics. The antibiotics were used at the following concentrations: streptomycin (50 μg/mL), ampicillin (100 µg/mL), and kanamycin (50 μg/mL).

### Construction of plasmids

Cloning was performed using the Gibson assembly method (Gibson et al., 2008; Gibson et al., 2009). To label Tir with V5, the pSA10 plasmid was amplified using the pSA10_vector_F/pSA10_vector_R primer pair and the Tir gene was amplified from EPEC genomic DNA and fused to a V5 tag at the C terminus in two sequential reactions using the Tir_V5_F1/Tir_V5_R1 and then Tir_V5_F1/Tir_V5_R2 primer pairs (**Table S2**). The PCR products were subjected to digestion with *Dpn*I, purified, and assembled by the Gibson assembly method.

All TMD-exchanged versions of *tir* (**Figure 1A**) were generated using a similar method; Tir TMD1, Tir TMD2, and EscD TMD were amplified with the appropriate primers (**Table S2**) and fused to a PCR fragment of the 200 bp sequence downstream of the replaced TMD, using an overlapping PCR (**Table S2**). This fusing was required to prevent the degradation of the inserted sequence from the exonuclease enzyme found in the Gibson assembly mix. Gibson assembly was conducted by amplifying the pTir_wt_-V5 (pSA10) vector with the appropriate primer pair to open the vector at the site of the replaced TMD (**Table S2**), followed by *Dpn*I treatment of the reaction and ligation of the amplified vector and the desired TMD insert. To generate a Tir protein that contains TMDs of reverse order, we used a similar approach with minor modification; the Gibson assembly was conducted by amplifying the pTir_-D2_-V5 (pSA10) vector and ligating the PCR fragment of TMD1 fused to a 200 bp sequence downstream of the TMD2 (**Table S2**).

To label CesT with a His tag at its C terminus, the *cesT* coding region was amplified from EPEC genomic DNA using the CesT_His_F/CesT_His_R primer pair. The PCR product was then subcloned as an *Nco*I/*Xho*I fragment into *Nco*I/*Xho*I-digested, His-tagged pET28a. All constructs were verified by DNA sequencing.

### *In vitro* type III secretion (T3S) assay

T3S assays were performed as previously described (Mitrovic and Sal-Man, 2022). Briefly, EPEC strains were grown overnight in LB supplemented with the appropriate antibiotics in a shaker at 37°C. The cultures were diluted 1:40 into pre-heated Dulbecco’s modified Eagle’s medium (DMEM, Biological Industries) and grown statically for 6 h in a tissue culture incubator (with 5% CO_2_) to an optical density of 0.7 at 600 nm (OD_600_). To induce protein expression, 0.25 mM IPTG was added to the bacterial cultures. The cultures were then centrifuged at 20,000 × *g* for 5 min to separate the bacterial pellets from the supernatants, the pellets were dissolved in SDS-PAGE sample buffer, and the supernatants were collected and filtered through a 0.22-μm filter (Millipore). The supernatants were normalized according to the bacterial OD_600_ and then precipitated with 10% (v/v) trichloroacetic acid overnight at 4°C to concentrate the proteins secreted into the culture medium. The samples were then centrifuged at 18,000 × *g* for 30 min at 4°C, the precipitates of the secreted proteins were dissolved in SDS-PAGE sample buffer, and the residual trichloroacetic acid was neutralized with saturated Tris. The proteins were analyzed by western blotting.

### Western blot

Samples were subjected to SDS-PAGE and transferred to nitrocellulose membrane (pore size, 0.45 µm; Bio-Rad). The membranes were blocked for 1 h with 5% (w/v) skim milk/PBST (0.1% Tween in phosphate-buffered saline), incubated with the primary antibody (diluted in 5% skim milk/PBST) for 1 h at room temperature or overnight at 4°C, washed, and then incubated with the secondary antibody (diluted in 5% skim milk/PBST) for 1 h at room temperature. Chemiluminescence was detected with EZ-ECL reagents (Biological Industries). The following primary antibodies were used: mouse anti-His (Pierce), diluted 1:2,000; rabbit anti-MBP (Thermo Fisher Scientific), diluted 1:1,000; mouse anti-DnaK (Abcam, Inc.), diluted 1:5,000; and mouse anti-actin (MPBio), diluted 1:10,000. Antibodies directed against T3SS components were a generous gift from Prof. B. Brett Finlay (University of British Columbia, Canada) and Prof. Rebekeh DeVinney (University of Calgary, Canada) and included mouse anti-Tir, rat anti-Intimin, and rat anti-Tir. Horseradish peroxidase-conjugated (HRP)-goat anti-mouse (Abcam Inc.), HRP-conjugated goat anti-rabbit (Abcam Inc.), and HRP-conjugated goat anti-rat (Jackson ImmunoResearch), diluted 1:10,000, were used as the secondary antibodies. Representative western blots of at least three independent experiments are presented in the Results section.

### Bacterial fractionation

Bacterial cell fractionation was performed as previously described (Gauthier et al., 2003). Briefly, EPEC strains from an overnight culture were subcultured 1:50 in DMEM and grown statically for 6 h at 37°C in a CO_2_ tissue culture incubator. To induce protein expression, 0.25 mM IPTG was added to the bacterial cultures. The cells were harvested, washed in PBS, and resuspended in buffer A [50 mM Tris (pH 7.5), 20% (w/v) sucrose, 5 mM EDTA, protease inhibitor cocktail (Roche Applied Science), and lysozyme (100 µg/mL)] and incubated with rotation for 15 min at room temperature to generate spheroplasts. MgCl_2_ was then added to a final concentration of 20 mM, and samples were centrifuged for 10 min at 8,000 × *g*. The supernatants containing the periplasmic fractions were collected. The pellets, which contained the cytoplasm and membrane fractions, were resuspended in lysis buffer (20 mM Tris (pH 7.5), 150 mM NaCl, 3 mM MgCl_2_, 1 mM CaCl_2_, and 2 mM β-mercaptoethanol with protease inhibitors). All subsequent steps were carried out at 4°C. RNase A and DNase I (10 µg/mL) were added, and the samples were sonicated (Fisher Scientific, 3 × 15 s). Intact bacteria were removed by centrifugation (2,300 × *g* for 15 min), and the cleared supernatants containing cytoplasmic and membrane proteins were transferred to new tubes. To obtain the cytoplasmic fraction, the supernatants were centrifuged (Beckman Optima XE-90 Ultracentrifuge with an SW60 Ti rotor) for 30 min at 100,000 × *g* to pellet the membranes. The supernatants, containing the cytoplasmic fraction, were collected, and the pellets, containing the membrane fractions, were washed with lysis buffer and resuspended in 0.1 mL lysis buffer with 0.1% SDS. The protein content of all samples was determined using BCA (Cyanagen) before the addition of SDS-PAGE sample buffer with β-mercaptoethanol. Intimin, maltose-binding protein (MBP), and DnaK were used as markers of the membrane, periplasmic, and cytoplasmic fractions, respectively.

### Co-elution by nickel affinity chromatography

*E. coli* BL21, expressing His-tagged CesT or one of the various Tir-V5 variants, from an overnight culture were subcultured 1:50 in 50 mL LB broth and grown with shaking for 6 h at 37°C. Two h post-inoculation, then, 0.25 mM IPTG was added to induce protein expression. Bacterial cells were collected by centrifugation (3,200 × *g* for 30 min) and resuspended in lysis buffer. The samples were sonicated (3 × 15 s; Fisher Scientific) and then incubated on ice for 15 min with 0.1% NP-40 (v/v). Intact bacteria were removed by centrifugation (18,000 × *g* for 15 min) and protease inhibitor cocktail was then added to the cleared supernatants. The samples were incubated rotating, at various combinations, with Ni-NTA resin at 4°C overnight. Finally, the nickel beads were collected by centrifugation (500 × *g* for 1 min) and washed five times with lysis buffer containing 10 mM imidazole. Proteins were eluted by the addition of sample buffer and boiling of the samples for 10 min. Whole cell lysates and eluted samples were analyzed using SDS- PAGE and western blot.

### Translocation activity

Translocation assays were performed as previously described (Baruch et al., 2011). Briefly, 3 × 10^6^ HeLa cells were infected for 3 h at a multiplicity of infection (MOI) of ~50 with EPEC strains that were grown statically overnight. The infected cells were then washed in cold PBS, collected, and lysed in designated lysis buffer [PBS with 0.5% Triton X-100 (v/v), protease inhibitor, and 1 mM DTT]. Thereafter, samples were centrifuged at 18,000 × *g* for 2 min to remove non-lysed cells, and supernatants were collected, boiled with SDS-PAGE sample buffer, and subjected to western blot with anti-Tir and anti-actin (loading control) antibodies. Uninfected samples and samples infected with the Δtir* mutant strain were used as negative controls.

To purify the cellular membranes of infected cells, washed cells were collected by scraping the plates. The cells were then resuspended in lysis buffer (3 mM imidazole [pH 7.4], 250 mM sucrose, 0.5 mM EDTA, and protease inhibitor) and mechanically fractionated by vigorous passage through a 25-G needle. The cellular lysates were centrifuged (3,000 × *g* for 15 min) to remove non-lysed cells and then further centrifuged (in a Beckman Optima XE-90 Ultracentrifuge with an SW60 Ti rotor at 100,000 × *g* for 40 min) to separate the cytoplasmic and membrane fractions.

### Gentamicin protection assay

HeLa cells (4.4 × 10^4^), seeded in a 96-well plate, were infected with EPEC strains (carrying a plasmid encoding carbenicillin resistance) at a MOI of 20 for 3 h. IPTG (0.25 mM) was added 0.5 h post-inoculation to induce protein expression. The cells were then washed with PBS and incubated with fresh DMEM containing gentamicin (100 μg/mL) for 1 h. The cells were then washed and lysed with PBS containing 0.1% Triton X-100 (v/v). Samples were collected and plated, at various dilutions, on LB agar plates containing carbenicillin. The plates were incubated overnight at 37°C and the numbers of colony-forming units (CFUs) were counted.

### Lactate dehydrogenase cytotoxicity assay

HeLa cells (5 × 10^4^ cells) were infected with pre-induced EPEC strains that were grown in DMEM statically for 3 h at 37°C in a CO_2_ tissue culture incubator with 0.25 mM IPTG. The infection was conducted at a MOI of 50 for 4 h. The culture supernatants were then collected and subjected to a CytoTox96 Non-Radioactive Cytotoxicity Assay (Promega) to determine their lactate dehydrogenase (LDH) levels. The absorbance at 490 nm was measured and calculated as the percentage of uninfected cells treated with the kit lysis buffer.

## Results

### Replacement of Tir TMD sequences alters protein secretion

We previously found that the TMDs of two TMD-containing secreted proteins are critical to promote protein secretion and prevent protein integration into the bacterial membrane (Gershberg et al., 2021). To examine whether Tir TMD sequences are also critical for protein secretion, we cloned Tir labeled with a V5 tag at its C terminus (pTir_wt_-V5) into a plasmid and created three Tir TMD variants with an alternative hydrophobic sequence. To minimize protein modification, we used the original TMDs of Tir, instead of unrelated TMD sequences, to create: (i) Tir-_D2_-V5, where the central 20 residues of the N-terminal TMD (TMD1) were replaced by the 20 central residues of the C-terminal TMD (TMD2) to form a variant with double TMD2, which we therefore named D2, (ii) Tir-_D1_-V5, where TMD2 was replaced by TMD1 to form a variant with double TMD1, which we therefore named D1, and (iii) Tir-_R21_-V5, where the Tir TMDs were in a reverse orientation, which we therefore named R21 (Tir variants are depicted in **Figure 1A**). In addition, we created two additional Tir TMD variants, where the TMDs were replaced by the TMD sequence of a structural membrane protein called EscD, which is integrated into the bacterial membrane and is part of the T3SS complex. To that end, we generated two Tir TMD-exchanged variants: Tir-_EscD1_-V5 (in which TMD1 is replaced by EscD TMD) and Tir-_EscD2_-V5 (in which TMD2 is replaced by EscD TMD), as depicted in **Figure 1A**.

Tir_wt_-V5 and the TMD-exchanged variants were transformed into EPEC Δtir*, grown under T3SS-inducing conditions, and examined for their ability to secrete Tir. We observed that WT EPEC secretes significant levels of Tir while the Δ*escV* mutant strain, with a deletion of one of the T3SS structural genes, expressed Tir but was unable to secrete it (**Figure 1B**). Note that the full-length Tir protein runs next to the 75 kDa marker, and shorter degradation bands are also observed. As expected, we did not detect Tir expression in EPEC Δtir* mutant. Nevertheless, transformation of this strain with pTir_wt_-V5 restored Tir expression and secretion (**Figure 1B**). Interestingly, Δtir* strains expressing Tir variants with replacement of TMD1 (Tir-_D2_-V5 and Tir-_EscD1_-V5) showed similar Tir secretion to EPEC WT, whereas strains expressing Tir variants with replacement of the TMD2 of Tir (Tir-_D1_-V5 and Tir-_EscD2_-V5) showed reduced Tir secretion (**Figure 1B**). The Δtir* strain expressing Tir-_R21_-V5 showed a similar secretion phenotype to the TMD2-exchanged variant (**Figure 1B**). The reduction in Tir secretion in these latter strains (Tir-_D1_-V5, Tir-_R21_-V5, and Tir-_EscD2_-V5) was accompanied by Tir enrichment at the bacterial pellets (**Figure 1B**), suggesting that Tir secretion was altered but not its overall expression. Tir is an effector protein; therefore, it should mainly be secreted by the T3SS at the late stage, following attachment to the host cells (Deane et al., 2010; Gaytan et al., 2016). This regulation prevents the premature secretion of effectors into the extracellular environment and was suggested to be sensed by a drop in calcium concentration (Shaulov et al., 2017). To simulate the host-cell attachment, in the absence of cells, bacteria can be transferred to calcium-free DMEM medium to enhance effector secretion. Examination of Tir secretion under these conditions revealed a similar secretion pattern as under regular T3SS-inducing conditions (**Figure S1**), further validating our results that alteration of Tir TMD2 interferes with the ability of the protein to be secreted by the T3SS.

### The TMD sequences of Tir influence the protein subcellular localization

**Figure 2:**
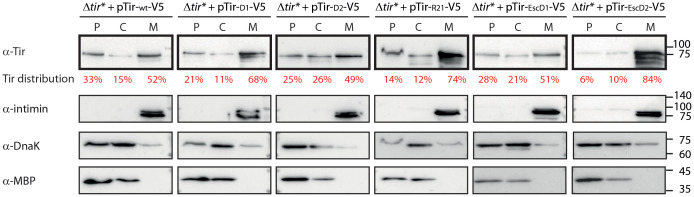
Replacement of TMD2 leads to altered Tir localization and enhanced bacterial membrane localization. EPEC Δtir* strain expressing Tir_wt_-V5 or TMD-exchanged Tir variants were grown in calcium-free DMEM to simulate T3SS effector secretion. The bacterial pellets were collected, lysed, and fractionated into periplasmic (P), cytoplasmic (C), and membrane (M) fractions. The samples were loaded on SDS-PAGE and analyzed by western blot using an anti-Tir antibody (blots are marked in bold frames). Tir levels were quantified by a densitometer, and the percentage of Tir at each fraction is presented in red font under the anti-Tir western blot. To confirm correct bacterial fractionation, western blots were also probed with anti-MBP (periplasmic marker), anti-DnaK (cytoplasmic marker), and anti-intimin (membrane marker) antibodies and presented below each bacterial strain.

The correlation between the reduced secretion of TMD2-exchanged Tir variants and their enhanced levels within the bacterial pellets suggested that these variants are mislocalized. To examine this aspect, we grew the Tir variant strains under T3SS-inducing conditions. Whole cell extracts were fractionated to obtain cytoplasmic, periplasmic, and membrane fractions. Evaluation of western blot with anti-Tir antibody revealed that the TMD2-exchanged versions (Tir-_D1_-V5, Tir-_R21_-V5, and Tir-_EscD2_-V5) showed enriched localization to the membrane fraction, of ~70-80% of total bacterial Tir, compared with Tir_wt_-V5 and its TMD1-exchanged versions, which had ~50% of total bacterial Tir (**Figure 2**). Surprisingly, although the Tir-_EscD1_-V5 variant contains the only TMD sequence of a membrane protein, it did not dramatically alter the localization of Tir to the bacterial membrane. Correct bacterial fractionation was confirmed by western blots probed with anti-DnaK (a cytoplasmic marker), anti-MBP (a periplasmic marker), and anti-Intimin (a membrane marker) antibodies (**Figure 2**). Overall, the fractionation results suggested that TMD2 of Tir is more critical than TMD1 in targeting Tir for secretion and preventing it from bacterial membrane integration *via* the co-translational-translocation mechanism.

### Tir TMDs exchange does not affect CesT binding

**Figure 3:**
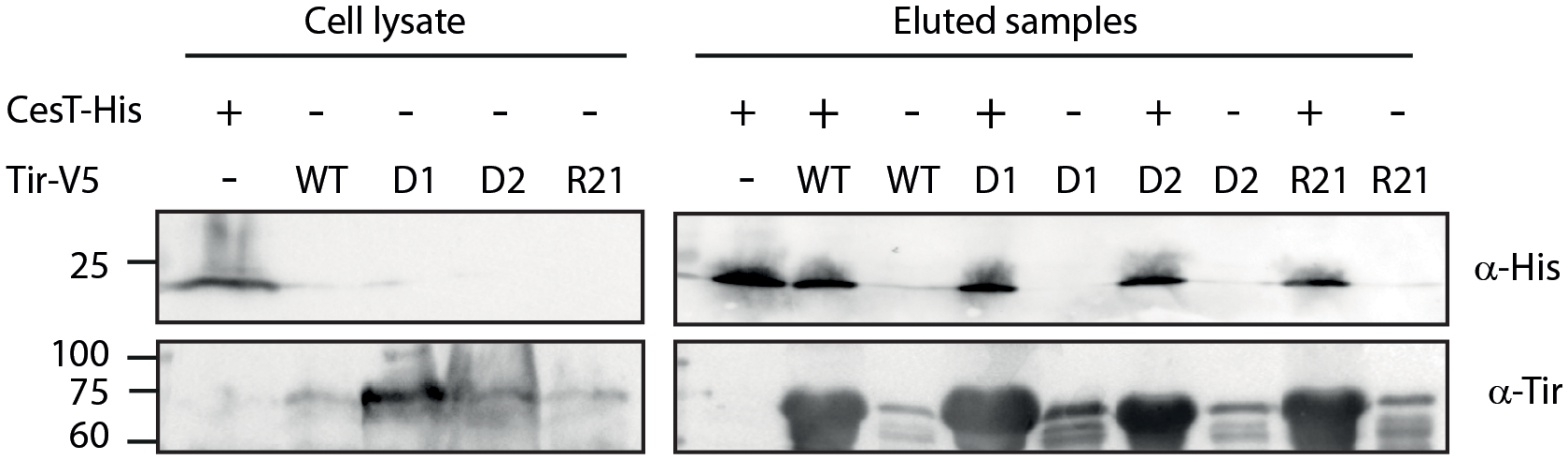
The Tir–CesT interaction is not disrupted by the replacement of Tir TMDs. *E. coli* BL21 (DE3) expressing Tir_wt_-V5, Tir-_D1_-V5, Tir-_D2_-V5, Tir-_R21_-V5, or CesT-His were grown for 6 h. The bacterial cells were collected, lysed, mixed, and incubated overnight with nickel beads. Bacterial lysates and eluted fractions were loaded on a 12% SDS-PAGE gel and analyzed by western blotting with anti-His and anti-Tir antibodies.

To examine whether the replacement of Tir TMD sequences disrupted the Tir–CesT interaction, we expressed WT and TMD-exchanged Tir variants in *E. coli* BL21 to prevent Tir secretion, as well as His-tagged CesT, lysed the bacteria, incubated the mixed lysates with nickel beads, and pulled down CesT-His together with its binding partners. We examined only the Tir variants containing Tir native sequences to minimize background noise that might occur due to non-specific EscD interactions. SDS-PAGE and western blot analysis of the eluted samples, using anti-His and anti-Tir antibodies indicated that all Tir variants co-eluted with CesT-His to a similar extent as WT Tir (**Figure 3**). Incubation of the Tir lysates in the absence of CesT-His resulted in minor non-specific binding to the nickel beads, which was similar across all Tir variants. Taken together, these results suggested that replacement of the TMDs did not disrupt the interaction between CesT and Tir.

### Tir TMDs affect protein translocation into host cells and plasma membrane integration

**Figure 4:**
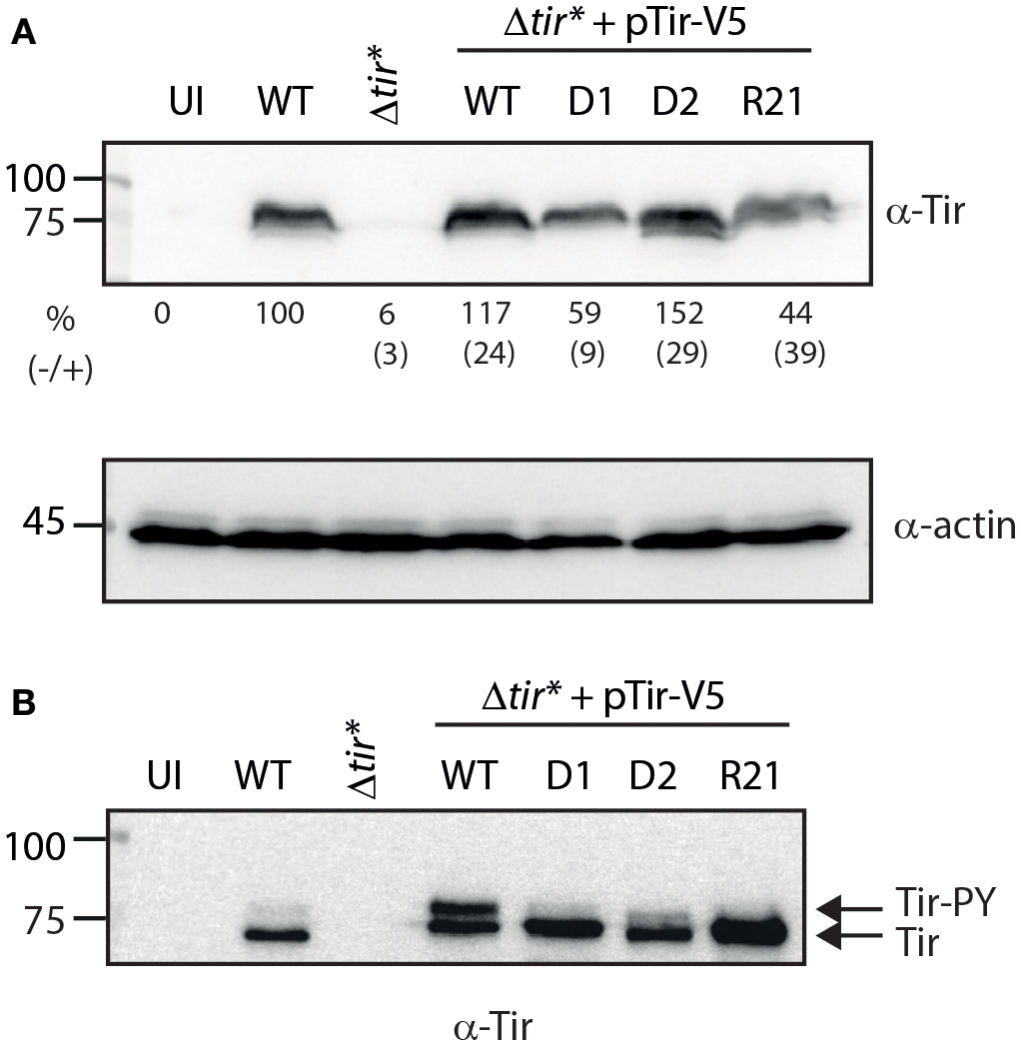
Replacement of TMD2 reduces the levels of phosphorylated Tir within the membrane fraction. **(A)** HeLa cells were infected with WT, Δtir*, and EPEC Δtir* expressing Tir_wt_-V5, Tir-_D1_-V5, Tir-_D2_-V5, or Tir-_R21_-V5 for 3 h or left uninfected (UI). The cells were washed and lysed using PBS with Triton X-100. Cell lysates were subjected to western blot with anti-Tir and anti-actin (control) antibodies. The average values and standard errors of Tir translocation level as a percentage of translocated Tir observed for WT EPEC (from three independent experiments) are presented along a representative blot. **(B)** The membrane fractions of HeLa cells infected with the various bacterial strains were subjected to western blot with anti-Tir antibody.

To examine Tir variants in a system that better simulates the natural process of the Tir protein, we infected host cells with bacteria expressing WT or TMD-exchanged Tir and examined their ability to properly translocate Tir into the cells. High levels of Tir were detected in EPEC WT cells, whereas no Tir was detected in uninfected cells or cells infected with the EPEC Δtir* mutant strain (**Figure 4A**). Importantly, we detected similar levels of translocated Tir in cells infected with EPEC Δtir* expressing WT Tir (pTir_wt_-V5) and in cells infected with WT EPEC (**Figure 4A**). While EPEC Δtir* expressing Tir-_D2_-V5 showed a similar translocation level to WT EPEC and EPEC Δtir* expressing Tir_wt_-V5, EPEC Δtir* expressing Tir-_D1_-V5 and Tir-_R21_-V5 showed reduced levels of translocated Tir in the infected cells (**Figure 4A**). The average values and standard errors of Tir translocation level as a percentage of translocated Tir observed for WT EPEC (from three independent experiments) are presented under the western blot of anti-Tir (**Figure 4A**). Actin was used as a loading control.

Because Tir needs to be presented on the host membrane to allow bacterial adhesion and pedestal formation, we further separated the infected cells into soluble and membrane fractions. In addition, because Tir phosphorylation in the membrane was suggested to be required for efficient actin polymerization, we assessed the level of Tir phosphorylation in the membrane by subjecting the membrane samples to low-percentage acrylamide gels that can separate unphosphorylated (~75 kDa) and phosphorylated (~90 kDa) Tir. Samples of HeLa cells infected with EPEC WT exhibited a high level of unphosphorylated Tir and a low level of phosphorylated Tir. This phenotype was enhanced in samples infected with EPEC Δtir* expressing Tir_wt_-V5, probably due to Tir overexpression (**Figure 4B**). Membrane samples of HeLa cells infected with EPEC Δtir* expressing Tir-_D1_-V5 and Tir-_R21_-V5 showed relatively similar overall levels of Tir to the membranes of HeLa cells infected with EPEC Δtir* expressing Tir_wt_-V5, but with no apparent detection of phosphorylated Tir (**Figure 4B**). The membranes of HeLa cells infected with EPEC Δtir* expressing Tir-_D2_-V5 showed a slightly reduced level of translocated Tir but had a low level of phosphorylated Tir (**Figure 4B**). These results suggested that alteration of the TMD sequences of Tir had a much milder effect on the translocation ability of Tir relative to the effect that we observed using Tir as a model system for protein secretion.

### The second TMD sequence of Tir is involved in bacterial invasiveness

**Figure 5:**
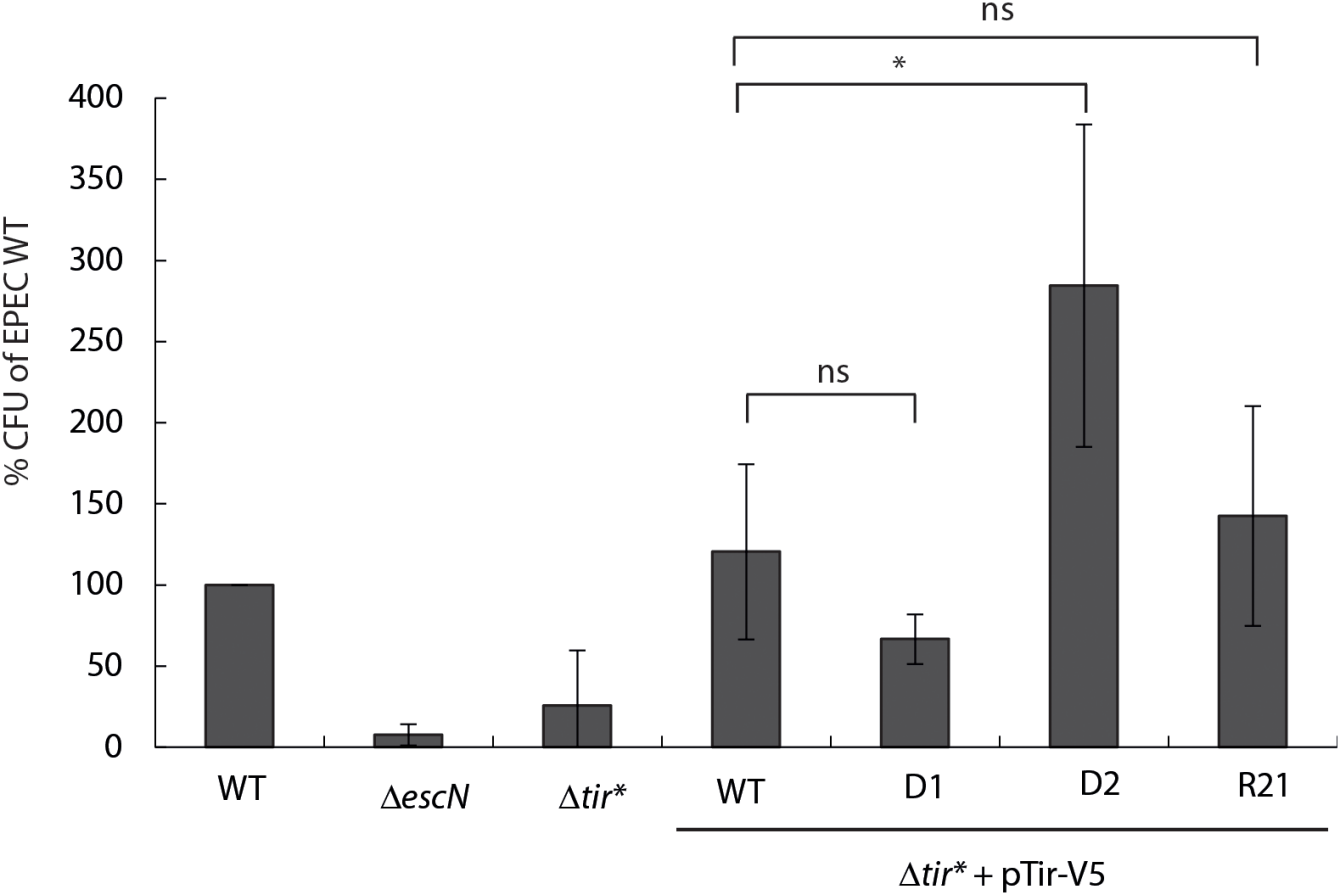
Gentamicin protection assay. HeLa cells were infected with EPEC WT, Δ*escN*, Δtir*, and EPEC Δtir* expressing Tir_wt_-V5, Tir-_D1_-V5, Tir-_D2_-V5, or Tir-_R21_-V5 for 3 h. The cells were washed and incubated in fresh DMEM with 100 µg/mL gentamicin for 1 h. The cells were then washed and lysed with Triton X-100 (0.1% (v/v)). Samples were then plated at serial dilutions on LB plates with carbenicillin. The plates were incubated overnight at 37°C, and bacterial colony-forming units (CFUs) were then counted. The percent of bacterial colonies relative to EPEC WT is presented. Bars represent geometric means for each strain, tested in triplicate in five independent experiments. Error bars represent standard deviation. *, p<0.05, unpaired Student’s t-test. ns, not significant.

To examine the role of Tir TMDs in proper protein folding and function, we performed a gentamicin protection assay. Although EPEC is generally considered an extracellular pathogen, it possesses limited invasive abilities both *in vivo* and *in vitro* (Yamamoto et al., 2009; Lai et al., 2013). This bacterial invasiveness has been linked to Tir–intimin-mediated adherence, which sometimes manifests in the localization of Tir to the intra-vacuolar bacterium (Bulgin et al., 2009), suggesting that the invasiveness of EPEC can indicate Tir functionality. To that end, HeLa cells were infected with the various Tir-exchanged variant strains for 3 h and then incubated for 1 h with gentamicin. The cells were then washed and lysed, and the lysates were plated on LB plates for overnight incubation. We determined the CFUs and calculated them as a percentage of the WT EPEC count (**Figure 5**). As expected, we detected significantly lower CFUs for the Δ*escN* mutant strain, which lacks the T3SS ATPase and therefore has no T3SS activity, and for Δtir* compared with WT EPEC. Transformation of the Δtir* with pTir_wt_-V5 restored the CFUs to the same level as that of WT EPEC, indicating proper functionality of Tir. The strains expressing the TMD2-exchanged versions (Tir-_D1_-V5 and Tir-_R21_-V5) yielded CFU levels that were lower than or similar to those of WT, but the Δtir* expressing Tir-_D2_-V5 yielded significantly higher CFUs compared with WT EPEC (**Figure 5**), thereby suggesting that Tir-_D2_-V5 protein induces significantly higher invasiveness activity, by altering Tir clustering, Tir-Intimin interactions, or the interaction of Tir with host-cellular proteins. To confirm that the enhanced invasiveness observed for Δtir* strain expressing Tir-_D2_-V5 did not result from its increased growth in the cellular infection medium, we assessed the bacterial growth before adding gentamicin. Samples were collected and plated on LB plates for overnight incubation, and their CFUs were measured. All tested strains demonstrated similar CFUs, indicating that enhanced invasiveness of Δtir* expressing Tir-_D2_-V5 was not due to improved growth (**Figure S2**). In addition, we examined the bacterial adhesion of the strains and found Tir variants show similar cellular adherence (**Figure S3**).

### Tir TMDs are involved in the cytotoxicity induced by EPEC

**Figure 6:**
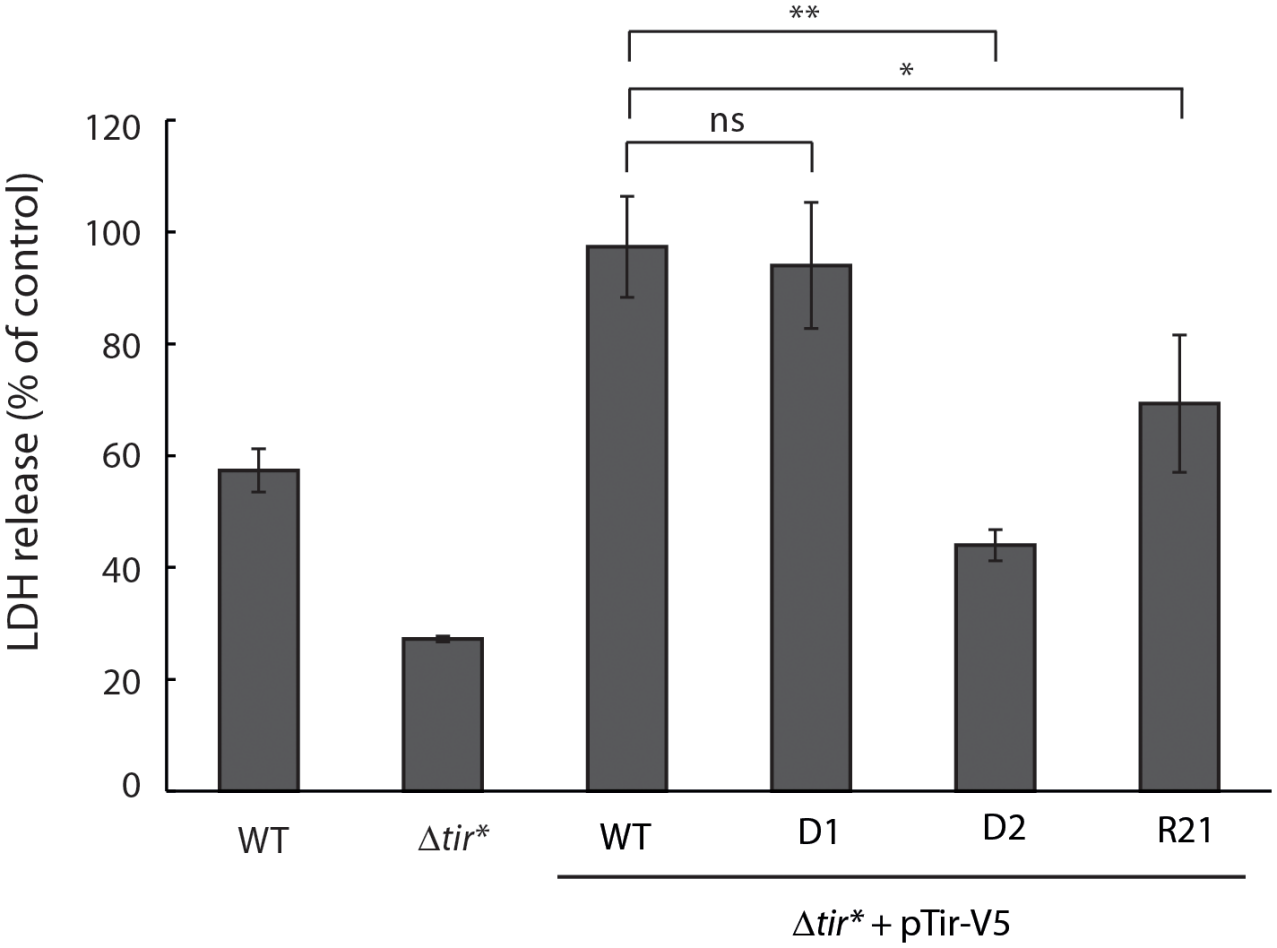
Overexpression of Tir increases cellular damage while replacement of TMD1 reduces cellular damage. EPEC WT, Δtir*, and EPEC Δtir* expressing Tir_wt_-V5, Tir-_D1_-V5, Tir-_D2_-V5, or Tir-_R21_-V5 variants were grown under T3SS-inducing conditions for 3 h before the infection of HeLa cells for 4 h. The culture supernatants were then collected and analyzed for LDH release. Bars represent geometric means for each strain, tested in quintuplicates over three independent experiments. Error bars represent standard deviation. **, p < 0.01; *, p<0.05, unpaired Student’s t-test. ns, not significant.

To further investigate the role of Tir TMDs in the postsecretion function of Tir, we assessed the release of LDH following the infection of cells with bacteria expressing WT or TMD-exchanged Tir variants. This method has been widely used in a variety of cell lines to evaluate EPEC-induced cell death, which is linked to intimate adherence and the interception of apoptotic pathways (Spitz et al., 1995; Crane et al., 1999; Samba-Louaka et al., 2009; Shames et al., 2010). Specifically, an LDH cytotoxicity assay was recently used to demonstrate the involvement of Tir phosphorylation and its interaction with intimin in the induction of pyroptosis *via* NLRP3 inflammasome activation in THP-1 cells (Goddard et al., 2019). In addition, the clustering of Tir during EPEC infection was shown to induce cell death (Zhong et al., 2020). To that end, we grew the bacteria under T3SS-inducing conditions and used them to infect HeLa cells for 4 h. The culture supernatants were then collected and the LDH levels were compared with those of lysed cells. Infection with EPEC WT showed LDH release that indicated about 60% cellular toxicity while the Δtir* mutant showed significantly less LDH release, about 30% (**Figure 6**). Unexpectedly, infection with Δtir* mutant expressing Tir_wt_-V5 resulted in significantly higher levels of LDH release, which reached the maximal value (**Figure 6**). These findings suggested that overexpression of Tir increases cellular cytotoxicity. Surprisingly, while infection with the TMD2-exchanged Tir variants (Tir-_D1_-V5 and Tir-_R21_-V5) resulted in similar or slightly lower released LDH levels compare to Tir_wt_-V5, infection with the Tir-_D2_-V5 variant resulted in a significant decrease in LDH release (~45%) (**Figure 6**). These results suggested that the cytotoxic effect induced by Tir clustering within the host membranes (Zhong et al., 2020), is linked to Tir TMDs because either replacement of TMD1 or duplication of TMD2 reduced the cytotoxic response of the cells to bacterial infection.

## Discussion

Tir is an effector protein that is translocated into host cells *via* the T3SS. After translocation, Tir is inserted across the apical plasma membrane, in a yet-to-be-elucidated mechanism, binds intimin, and sequentially activates a cellular cascade resulting in actin modulation. Tir belongs to the unique family of TMD-containing secreted proteins, which manage to escape integration into the bacterial membrane and get inserted into the host plasma membrane instead. Previous research has demonstrated that the TMDs of T3SS translocators are important for the ability of the protein to be secreted and for their postsecretion functions (Gershberg et al., 2021).

In this study, we examined whether this observation represents a more general phenomenon, common to many TMD-containing secreted proteins. Moreover, we studied whether the context of the TMDs in the protein sequence affects protein secretion and function. To that end, we chose Tir protein, which is a secreted effector that contains two TMDs, and cloned WT and TMD-exchanged variants. We first assessed the effects of the exchanged TMDs on the ability of Tir to be secreted through the T3SS complex. Interestingly, we observed reduced secretion of all Tir variants that contained a TMD2 exchange, whereas replacement of TMD1 was associated with normal secretion (**Figure 1B**). These results suggested that the second TMD of Tir is essential for the secretion of Tir. The fact that the reduced secretion of TMD2-exchanged variants was accompanied by enrichment of Tir at the bacterial pellets (**Figure 1B**) suggested that its secretion was affected by the TMD exchange but not its expression. Because TMDs are involved in the targeting of proteins to the membrane and can therefore interfere with protein secretion, we examined the subcellular localization of Tir variants. We observed that the TMD2-exchanged variants, which showed reduced secretion, were enriched in the bacterial membrane compared with Tir_wt_-V5 and TMD1-exchanged variants (**Figure 2A**). This result is in line with our previous report showing that replacement of the translocator TMDs with an alternative hydrophobic sequence resulted in their mislocalization into the bacterial membrane (Gershberg et al., 2021). Interestingly, the Tir-_EscD1_-V5 variant did not display enriched membrane localization, despite containing the only TMD sequence of an actual membrane protein. Taken together, these results further demonstrate that TMD2 of Tir is involved in determining the destination of Tir, probably by promoting the escape of the protein from the bacterial membrane integration mechanism.

As previously mentioned, Tir and other secreted proteins interact with chaperones to prevent their premature folding and erroneous integration into the bacterial membrane and to lead them to the injectosome for secretion. To examine whether the mislocalization of Tir variants results from impaired interaction with the Tir chaperone CesT, we examined CesT–Tir interaction using affinity chromatography. The results indicated that all Tir variants co-eluted with CesT-His to a similar level as Tir_wt_-V5 (**Figure 3**), thereby suggesting that TMD replacement did not disrupt Tir–CesT binding. Nevertheless, because both Tir and CesT bind the T3SS ATPase EscN directly and in complex (Gauthier and Finlay, 2003), it is possible that, while the TMDs are not directly involved in the Tir–CesT interaction, they are involved in the interactions within the CesT–Tir–EscN complex or the dissociation kinetics between CesT and Tir to allow Tir secretion.

It was previously suggested that the moderate hydrophobicity of the TMDs is a critical factor in the targeting of TMD-containing secreted proteins for secretion (Krampen et al., 2018). In an attempt to explain the different phenotypes observed by the replacement of each TMD, we calculated the apparent free energy differences (ΔG_app_) required for TMD insertion using Jpred (Hessa et al., 2007). A negative ΔG_app_ value predicts its recognition as a TMD helix and its membrane integration, whereas a positive value does not exclude membrane integration yet suggests the need for a stabilizing interaction (Hessa et al., 2007). The calculated ΔG_app_ values for TMD1 and TMD2 of Tir were 0.938 and 2.385, respectively. While both values are positive, the ΔG_app_ for TMD2 was significantly higher. Because proteins with relatively low calculated ΔG_app_ are targeted to the bacterial membrane (e.g., the calculated ΔG_app_ for the TMD of EscD is 0.123), the high ΔG_app_ of Tir TMD2 might explain the different contributions of TMD1 and TMD2 regarding the secretion phenotype of Tir. Interestingly, our results demonstrated that, even though the Tir-_R21_-V5 variant contains the original WT sequence, the switched positions of the TMDs affected protein secretion. These results suggested that the location of the TMDs, and not exclusively their sequence, affects their contribution to protein secretion. This was further supported by the observation that, while Tir with the double TMD1 sequence (Tir-_D1_-V5) did not support full Tir secretion in this study, replacement of the TMD of EspB with TMD1 of Tir resulted in similar EspB secretion as WT EspB (Gershberg et al., 2021). These results suggested that the TMD sequence by itself cannot stimulate protein secretion, but rather the TMD within the specific context of the targeted protein. Altogether, these results support our conclusion of the involvement of the TMD sequence in the secretion process of TMD-containing secreted proteins and suggest that the context, and not only the presence of TMD per se, within the protein sequence is also critical.

While we can use Tir to study TMD-containing secreted proteins, it is worth mentioning that Tir is an effector that normally does not get secreted to the extracellular space. Using the T3SS assay, we created segregation between the secretion and translocation phases of Tir, which does not occur naturally. Hence, while this method allows us to assess the role of Tir TMDs in the secretion process, these results are not necessarily indicative of the involvement of TMDs in Tir translocation and postsecretion functions. Therefore, to study the involvement of Tir TMDs in its ability to translocate into host cells, we infected HeLa cells with EPEC Δtir* expressing Tir_wt_-V5 and TMD-exchanged variants. We observed that, while all Tir variants translocated into the host cells, the TMD2-exchanged versions (Tir-_D1_-V5 and Tir-_R21_-V5) exhibited lower translocation levels (**Figure 4A**). Membrane fractionation of the cells revealed that all Tir variants translocated into the host membrane, although the TMD2-exchanged variants were poorly phosphorylated (**Figure 4B**). A possible explanation of these results is that the TMD2-exchanged variants orient incorrectly across the membrane or interact insufficiently with intimin to promote phosphorylation. To link these results with protein function, we performed a gentamicin protection assay that evaluates bacterial invasiveness as a means to determine the Tir–intimin interaction (Bulgin et al., 2009). We found that expression of Tir_wt_-V5 in EPEC Δtir* restored bacterial invasiveness to EPEC WT levels, thus confirming that EPEC invasiveness is Tir-dependent (**Figure 5**). Surprisingly, strains expressing TMD2-exchanged versions yielded CFUs that were not significantly different from those of WT EPEC, not supporting our hypothesis that these Tir variants are folded incorrectly or interact insufficiently with intimin. In addition, LDH release, which quantifies the cytotoxic effect linked to Tir phosphorylation and interaction with intimin, showed that infection with TMD2-exchanged variants resulted in a cytotoxic effect similar to that of EPEC Δtir* expressing Tir_wt_-V5 (**Figure 6**). This incompatibility between the Tir phosphorylation level and the functional assay suggests that either Tir phosphorylation is not critical for Tir activity or that a very low phosphorylation level is sufficient for Tir function. In addition, based on our results, we can conclude that the TMD2 sequence is not critical for Tir activity post-translocation.

Unexpectedly, we observed that Δtir* expressing Tir-_D2_-V5 showed very high invasiveness while inducing significantly less LDH release compared with WT EPEC or Δtir* expressing Tir_wt_-V5 (**Figures 5**, **6**). This negative correlation suggests that hyperinvasive EPEC is less virulent and therefore has a lower cytotoxic effect on host cells. In addition, the results indicated that TMD1 is critical for Tir activity and that its replacement with an alternative TMD sequence disrupted Tir function. Whether it was the lack of TMD1 or the addition of TMD2 that altered Tir function is yet to be determined. Because TMDs were previously shown to be involved in various protein–protein interactions and membrane organization (de Planque and Killian, 2003; Fink et al., 2012; Tseytin et al., 2018), it is possible that replacement of the TMD1 sequence with that of TMD2 altered Tir interactions with other proteins or changed the membrane properties. Regardless, we observed that TMD1 is more critical for Tir function than TMD2.

Overall, our results support our previous finding that the TMD sequences of TMD-containing secreted proteins are different from classical TMD sequences and that this allows them to escape the bacterial membrane integration mechanism. Moreover, these sequences appear to be defined not only by their hydrophobicity level, but also by their adjacent protein sequence. Furthermore, while Tir TMD2 is more critical for protein section, TMD1 is more critical for the activity of the protein within the host cells.

## Data availability statement

The raw data supporting the conclusions of this article will be made available by the authors, without undue reservation.

## Author contributions

DB - Conception and design of study, acquisition of data, analysis of data, writing the manuscript. JG - acquisition of data and analysis of data. NS-M - Conception and design of study, monitoring the experiments and data analysis, writing the manuscript. All authors contributed to the article and approved the submitted version.
