# Supplementary material for: The transmembrane domains of the type III secretion system effector Tir are involved in its secretion and cellular activities
Source: Front Cell Infect Microbiol. 2023 Feb 14;13:1103552. doi: 10.3389/fcimb.2023.1103552 (PMC9971567; doi:10.3389/fcimb.2023.1103552)
Supplement: Supplementary file 1 [file DataSheet_1.docx]

**Supplementary**


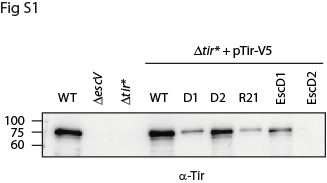


Figure S1. **Tir TMD2 is critical for protein secretion under conditions that stimulate the secretion of T3SS effectors.** Protein secretion profiles of EPEC strains grown in calcium-free DMEM medium that enhances effector secretion: WT, ∆*escV*, Δ*tir**, and Δ*tir** carrying the pTir_wt_-V5 and Tir TMD-exchanged variants. The secreted fractions were normalized, concentrated from the supernatants of bacterial cultures, and analyzed by western blotting with an anti-Tir antibody.


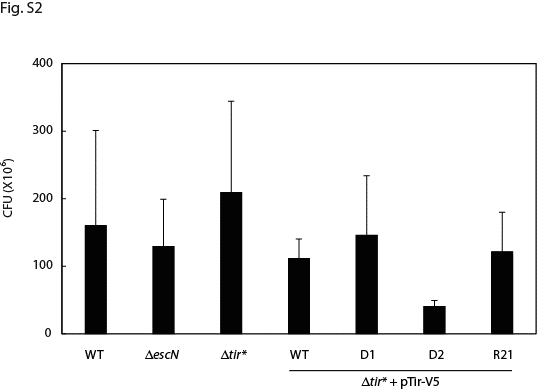


Figure S2. **Tir variants show similar growth in cellular infection medium.** EPEC WT, ∆*escN*, Δ*tir**, and Δ*tir** carrying the pTir_wt_-V5 and Tir TMD-exchanged variants were incubated with HeLa cells for 3 h. Samples were taken before adding gentamicin and plated at serial dilutions on LB plates with carbenicillin. The plates were incubated overnight at 37°C, and bacterial colony-forming units (CFUs) were counted. Bars represent geometric means for each strain, tested in triplicate in three independent experiments. Error bars represent standard deviation. No statistical significance was observed between the bacterial growth of WT EPEC and the various strains.


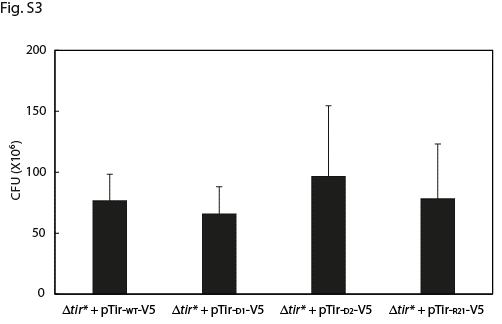


Figure S3. **Tir variants show similar cellular adherence.** EPEC Δ*tir** expressing Tir_wt_-V5, Tir-_D1_-V5, Tir-_D2_-V5, or Tir-_R21_-V5 were incubated with HeLa cells for 3 h, washed, and then plated at serial dilutions on LB plates with carbenicillin. The plates were incubated overnight at 37°C, and bacterial colony-forming units (CFUs) were counted. Bars represent geometric means for the CFU of each strain, tested in triplicate in two independent experiments. Error bars represent standard deviation. No statistical significance was observed between the various strains.

**Blots of Figure 1:**

Experiment #1:


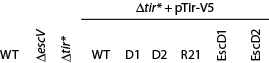


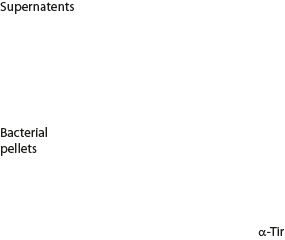

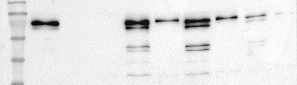


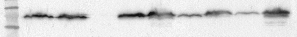


Experiment #2:


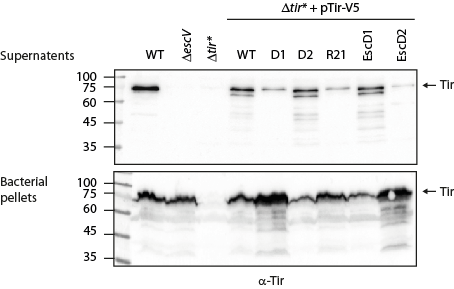


Experiment #3:


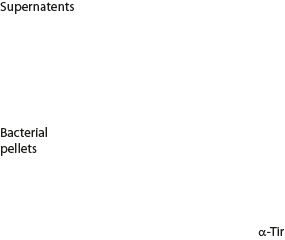

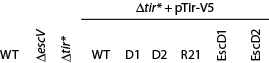

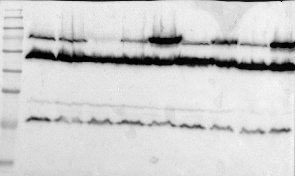

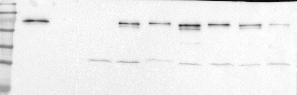


**Blots of Figure 2:**

Experiment #1:


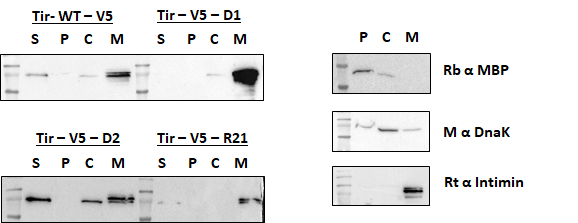


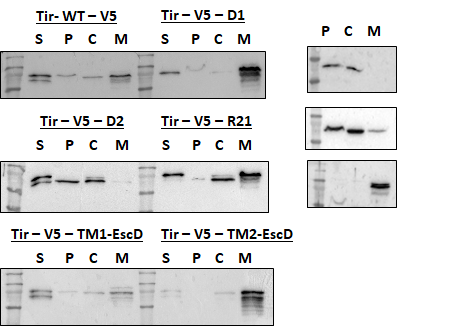
Experiment #2:

Experiment #3:


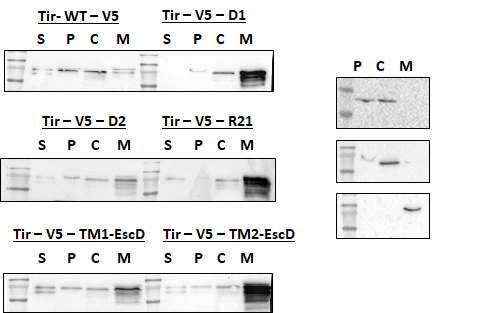


**Blots of Figure 3:**


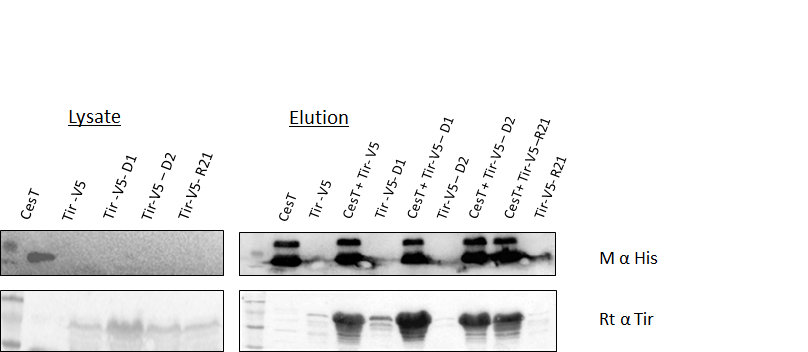
Experiment #1:

Experiment #2:


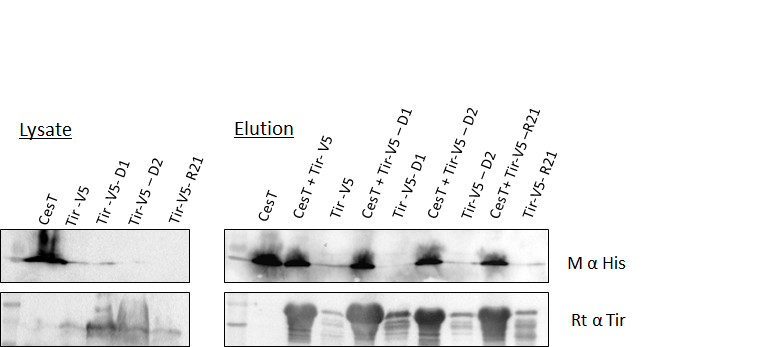


Experiment #3:


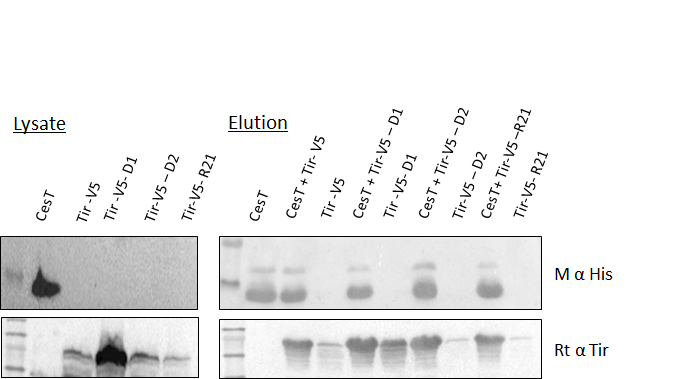


e

**Blots of Figure 4:**

Experiment #1:


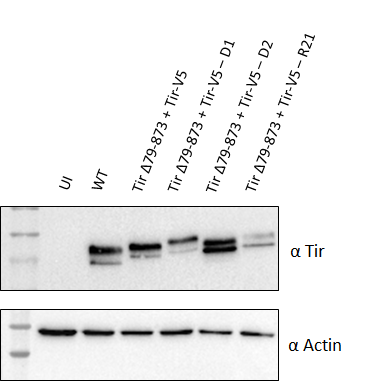


Experiment #2:


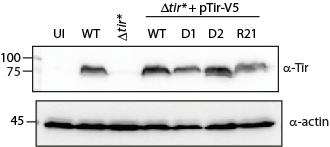


Experiment #3:


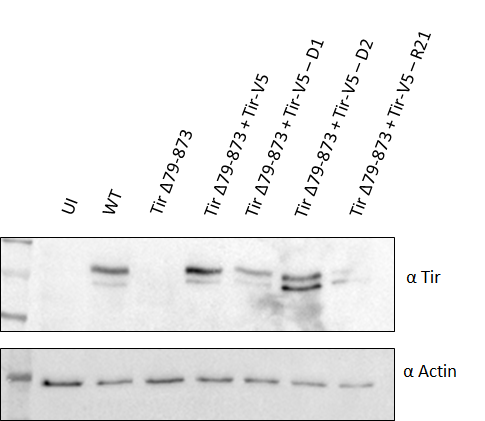


Table S1: Strains and plasmids used in this study

| **Strains** | **Description** | **Reference** |
| --- | --- | --- |
| Wild-type EPEC | EPEC strain E2348/69, streptomycin resistant | (Iguchi et al., 2009) |
| EPEC Δ*escN* | Non-polar deletion of *escN* | (Gauthier et al., 2003) |
| EPEC Δ*escV* | Non-polar deletion of *escV* | (Gauthier et al., 2003) |
| EPEC Δ*tir** | In-frame deletion of Tir 79-873 base pairs | (Elbaz et al., 2019) |
| *E. coli* DH10B | For plasmid handling | (Durfee et al., 2008) |
| *E. coli* BL21 (λDE3) | For protein expression |  |
| **Plasmids** |  |  |
| pTir_WT_-V5 (pSA10) | C-terminal V5-tagged Tir in pSA10 | This study |
| pTir-_D1_-V5 (pSA10) | C-terminal V5-tagged Tir with TMD1 sequence instead of TMD2 in pSA10 | This study |
| pTir-_D2_-V5 (pSA10) | C-terminal V5-tagged Tir with TMD2 sequence instead of TMD1 in pSA10 | This study |
| pTir-_R21_-V5 (pSA10) | C-terminal V5-tagged Tir with TMD1 sequence instead of TMD2 and vice versa in pSA10 | This study |
| pTir-_EscD1_-V5 (pSA10) | C-terminal V5-tagged Tir with EscD TMD sequence instead of TMD1 in pSA10 | This study |
| pTir-_EscD2_-V5 (pSA10) | C-terminal V5-tagged Tir with EscD TMD sequence instead of TMD2 in pSA10 | This study |
| pCesT-His (pET28a) | C-terminal His-tagged CesT in pET28a | This study |

Table S2: Sequences of primers designed and used in this study

| **Constructs and primers** | **primer sequence** |
| --- | --- |
| **pTir_WT_-V5 (pSA10)** |  |
| Tir_V5_F1 | ATTTCACACAGGAAACAGATGCCTATTGGTAACCTTGG |
| Tir_V5_R1 | CCGAGGAGAGGGTTAGGGATAGGCTTACCAACGAAACGTACTGGTCC |
| Tir_V5_R2 | CGGATCCCCGGGAATTTTACGTAGAATCCAGACCGAGGAGAGGGTTAG |
| pSA10_vector_F | AATTCCCGGGGATCCGTCG |
| pSA10_vector_R | CTGTTTCCTGTGTGAAATTGTTATCCG |
| **pTir-_D1_-V5 (pSA10)** |  |
| Tir_TMD1_F1 | GAGGAATTACAGCTTTCATCGTTCTGGGTTTCTGTCGGCG |
| Tir_TMD1_R1 | ATGGAGCGCAGTCGTAATACCAGTTGCCGCCAG |
| Tir_TMD2_200bp_F | GCAACTGGTATTACGACTGCGCTCCATAGACGAAATCAG |
| Tir_TMD2_200bp_R | CCCAACTTCAGCATATGGATTAACCACTTCGCTAGAGG |
| pSA10_Tir_TMD2_F | CCATATGCTGAAGTTGGGGG |
| pSA10_Tir_TMD2_R | CGATGAAAGCTGTAATTCCTCC |
| **pTir-_D2_-V5 (pSA10)** |  |
| Tir_TMD2_F1 | CGCTCAGATCCTAAAGGTATTGGTTACGGCCTC |
| Tir_TMD2_R1 | TGTCAAAGCCAACGCCTGTGCTACACCAGCACCAATTCC |
| Tir_TMD1_200bp_F | GCACAGGCGTTGGC |
| Tir_TMD1_200bp_R | CCTCTTTAGCTTGTTGTGCTATTTGCTCAACAATATCATC |
| pSA10_Tir_TMD1_F | ATAGCACAACAAGCTAAAGAGGC |
| pSA10_Tir_TMD1_R | TTTAGGATCTGAGCGAACGCTGG |
| **pTir-_R21_-V5 (pSA10)** |  |
| pSA10_Tir_TMD2_F | CCATATGCTGAAGTTGGGGG |
| pSA10_Tir_TMD2_R | CGATGAAAGCTGTAATTCCTCC |
| Tir_TMD1_F1 | GAGGAATTACAGCTTTCATCGTTCTGGGTTTCTGTCGGCG |
| Tir_TMD2_200bp_R | CCCAACTTCAGCATATGGATTAACCACTTCGCTAGAGG |
| **pTir-_EscD1_-V5 (pSA10)** |  |
| EscD_TMD_Tir_TMD1_F | CGCTCAGATCCTAAACTGGCGAGCGTGATTG |
| EscD_TMD_Tir_TMD1_R | GCCAACGCCTGTGCCAAAACGTAACTGCCGATG |
| Tir_TMD1_200bp_F | GCACAGGCGTTGGC |
| Tir_TMD1_200bp_R | CCTCTTTAGCTTGTTGTGCTATTTGCTCAACAATATCATC |
| pSA10_Tir_TMD1_F | ATAGCACAACAAGCTAAAGAGGC |
| pSA10_Tir_TMD1_R | TTTAGGATCTGAGCGAACGCTGG |
| **pTir-_EscD2_-V5 (pSA10)** |  |
| EscD_TMD_Tir_TMD2_F | GGAATTACAGCTTTCATCGCTGGCGAGCGTGATTG |
| EscD_TMD_Tir_TMD2_R | ATGGAGCGCAGTCGTCAAAACGTAACTGCCGATG |
| Tir_TMD2_200bp_F | ACGACTGCGCTCCATAGACGAAATCAG |
| Tir_TMD2_200bp_R | CCCAACTTCAGCATATGGATTAACCACTTCGCTAGAGG |
| pSA10_Tir_TMD2_F | CCATATGCTGAAGTTGGGGG |
| pSA10_Tir_TMD2_R | CGATGAAAGCTGTAATTCCTCC |
| **pCesT-His (pET28)** |  |
| CesT_His_F | GGCCATGGATGTCATCAAGATCTGAAC |
| CesT_His_R | GGCTCGAGTCTTCCGGCGTAATAATG |

**References:**

Durfee, T., Nelson, R., Baldwin, S., Plunkett, G., 3rd, Burland, V., Mau, B., Petrosino, J.F., Qin, X., Muzny, D.M., Ayele, M., Gibbs, R.A., Csorgo, B., Posfai, G., Weinstock, G.M., and Blattner, F.R. (2008). The complete genome sequence of *Escherichia coli* DH10B: insights into the biology of a laboratory workhorse. *J. Bacteriol.* 190**,** 2597-2606.

Elbaz, N., Socol, Y., Katsowich, N., and Rosenshine, I. (2019). Control of Type III Secretion System Effector/Chaperone Ratio Fosters Pathogen Adaptation to Host-Adherent Lifestyle. *mBio* 10.

Gauthier, A., Puente, J.L., and Finlay, B.B. (2003). Secretin of the enteropathogenic *Escherichia coli* type III secretion system requires components of the type III apparatus for assembly and localization. *Infect. Immun.* 71**,** 3310-3319.

Iguchi, A., Thomson, N.R., Ogura, Y., Saunders, D., Ooka, T., Henderson, I.R., Harris, D., Asadulghani, M., Kurokawa, K., Dean, P., Kenny, B., Quail, M.A., Thurston, S., Dougan, G., Hayashi, T., Parkhill, J., and Frankel, G. (2009). Complete genome sequence and comparative genome analysis of enteropathogenic *Escherichia coli* O127:H6 strain E2348/69. *J. Bacteriol.* 191**,** 347-354.
